# Utility of characters evolving at diverse rates of evolution to resolve quartet trees with unequal branch lengths: analytical predictions of long-branch effects

**DOI:** 10.1186/s12862-015-0364-7

**Published:** 2015-05-14

**Authors:** Zhuo Su, Jeffrey P Townsend

**Affiliations:** Department of Ecology and Evolutionary Biology, Yale University, New Haven, CT 06520 USA; Department of Biostatistics, Yale University, New Haven, CT 06520 USA; Program in Computational Biology and Bioinformatics, Yale University, New Haven, CT 06520 USA; Department of Biostatistics, Yale School of Public Health, 135 College St #222., New Haven, CT 06511 United States of America

**Keywords:** Long-branch effects, Felsenstein zone, Signal, Noise, Phylogenetic Inference

## Abstract

**Background:**

The detection and avoidance of “long-branch effects” in phylogenetic inference represents a longstanding challenge for molecular phylogenetic investigations. A consequence of parallelism and convergence, long-branch effects arise in phylogenetic inference when there is unequal molecular divergence among lineages, and they can positively mislead inference based on parsimony especially, but also inference based on maximum likelihood and Bayesian approaches. Long-branch effects have been exhaustively examined by simulation studies that have compared the performance of different inference methods in specific model trees and branch length spaces.

**Results:**

In this paper, by generalizing the phylogenetic signal and noise analysis to quartets with uneven subtending branches, we quantify the utility of molecular characters for resolution of quartet phylogenies via parsimony. Our quantification incorporates contributions toward the correct tree from either signal or homoplasy (*i.e.* “the right result for either the right reason or the wrong reason”). We also characterize a highly conservative lower bound of utility that incorporates contributions to the correct tree only when they correspond to true, unobscured parsimony-informative sites (*i.e.* “the right result for the right reason”). We apply the generalized signal and noise analysis to classic quartet phylogenies in which long-branch effects can arise due to unequal rates of evolution or an asymmetrical topology. Application of the analysis leads to identification of branch length conditions in which inference will be inconsistent and reveals insights regarding how to improve sampling of molecular loci and taxa in order to correctly resolve phylogenies in which long-branch effects are hypothesized to exist.

**Conclusions:**

The generalized signal and noise analysis provides analytical prediction of utility of characters evolving at diverse rates of evolution to resolve quartet phylogenies with unequal branch lengths. The analysis can be applied to identifying characters evolving at appropriate rates to resolve phylogenies in which long-branch effects are hypothesized to occur.

## Background

The detection and avoidance of long-branch effects in phylogenetic inference has been a longstanding challenge. Arising when there is unequal divergence among taxa, long-branch effects are caused by convergent and parallel changes that give rise to a systematic bias in the phylogenetic estimation procedure, producing one or more artefactual phylogenetic groupings of taxa [1-15]. While early investigations discussed long-branch effects as a significant problem for inference with parsimony, it has since been demonstrated that inference by maximum likelihood (ML) and Bayesian approaches can also be subject to long-branch effects [7-9,14-20], even when the correct model is specified exactly [11,21].

An extensive literature exists composed of simulation studies that have evaluated the performance of different inference methods on model trees, investigating the branch length conditions wherein long-branch effects lead to misleading results. For example, in what is classically termed the Felsenstein zone, two long-branched taxa are non-sisters in a four-taxon tree. Simulation studies have demonstrated that parsimony is more likely to group the long-branched non-sister taxa together (“long-branch attraction” [22-25]) than likelihood methods. Siddall [26] referred to the converse zone, where two long-branched taxa are true sisters in a four-taxon tree, as the “Farris zone”. Simulations performed by Swofford *et al.* [27] demonstrated that along a tree-axis that includes both the Felsenstein zone and the Farris zone, ML outperforms parsimony overall in recovering the correct quartet topology. Many subsequent simulation studies compared the performance of parsimony and ML in other model trees (*e.g.* [5,28-30]). As Bergsten [6] pointed out, the conclusions of these comparative simulation studies have been highly dependent on the specific model tree and branch length conditions subjectively chosen for individual investigations. Analysis of these comparative simulation studies shows clearly that parsimony has a strong bias towards grouping long-branched taxa together, but also that ML and other probabilistic methods that in principal account for unequal branch lengths and correct for unobserved changes [27,28] can minimize but not eliminate the risks of long-branch effects [6,31].

In contrast to the extensive simulation studies comparing the performance of different inference methods, few analytical frameworks are available to quantify the phylogenetic utility of molecular loci for resolving specific phylogenies with unequal branch lengths. Theory provided by Felsenstein [1], Hendy and Penny [2], and Kim [3] has revealed general branch length conditions in which inference becomes inconsistent. But because these works assume a character with binary states with equal substitution rates, the inconsistency conditions identified by assuming such a simplistic model cannot be directly applied to real-life molecular loci, which typically follow much more complex molecular evolutionary models and vary in rates of evolution.

Post-hoc analytical methods have been developed that detect the presence of long-branch effects in molecular data. For example, split decomposition [32] with spectral analysis [33] has been utilized to plot split graphs to show where conflicting signal exists in a molecular data set [10,34-38], and Relative Apparent Synapomorphy Analysis (RASA [39,40]) has been developed to detect problematic long branches by examining the taxon-variance plot of a molecular data set [41-49]. The taxon-variance plot has attracted some zealous criticism in several studies that report false outcomes for identifying problematic long branches [50-54]. No such method is perfect for all examples. Even so, one issue with these post-hoc analytical methods is that the graphic outputs produced evaluate realized sequence data to convey a qualitative sense rather than quantification of phylogenetic utility.

Recently, progress has been made towards analytical prediction of the utility of sequence data for resolving phylogenies in which long-branch attraction bias may arise. Extending the work of Fischer and Steel [55], which evaluated the sequence length needed for accurately resolving a binary four-taxon phylogenetic tree with four long subtending branches and a short internode, Martyn and Steel [12] investigated the required sequence length to resolve a quartet in which just one subtending branch is long, rather than all four, in the presence and absence of a molecular clock. However, they also demonstrated that those results were critically dependent on the assumption that all sites are evolving at a single rate. Susko [15] advanced an analytical method based on Laplace approximations to provide simple corrections for long-branch attraction biases in Bayesian-based inference towards particular topologies; the effectiveness of the corrections was further demonstrated in simulations of four-taxon and five-taxon trees.

In this paper, we quantify an accurate prediction of utility of molecular characters for resolving a quartet phylogeny with uneven subtending branches as assessed by parsimony, by incorporating contributions toward the correct tree from any parsimony-informative sites that are consistent with the actual quartet topology (*i.e.* support for the correct quartet topology due to true, unobscured signal or homoplasy). We also characterize a highly conservative lower bound of utility by incorporating contributions toward the correct tree only from those true, unobscured parsimony-informative sites (*i.e.* support for the correct topology due to true, unobscured signal only). We build on the signal and noise framework of Townsend et al. [56], which uses the estimated substitution rates of individual molecular characters to estimate the power of a set of molecular sequences for resolving a four-taxon tree with equal subtending branch lengths. This result, applied to the Poisson model of molecular evolution, was subsequently generalized by Su *et al.* [57] to apply to all standard symmetric molecular evolutionary models of nucleotide substitution up to and including the General Time Reversible model (GTR [58,59]). Herein we further generalize the signal and noise analysis by relaxing the assumption of equal subtending branch lengths for the four-taxon tree. Further, we use the generalized signal and noise analysis to explore how varying branch length conditions and alternative model assumptions affect the predicted phylogenetic utility. We apply the generalized signal and noise analysis to four-taxon trees in which long-branch attraction bias arises as a consequence of unequal evolution rates or an asymmetrical topology. We demonstrate that the generalized signal and noise analysis can help identify for these example phylogenies branch length conditions in which inference is inconsistent.

### Theory

#### Phylogenetic signal and noise

The Markov chain of a nucleotide character under the GTR model is commonly mathematically modeled by a four-by-four substitution rate matrix **Q**(*λ*), whose element *q*_*ij*_ gives the instantaneous rate at which the nucleotide character changes from nucleotide *i* to nucleotide *j*, where *j ≠ i,* and *i, j* = T, C, A, or G (*c.f.* Equation 1 in [57]). The average substitution rate of the character, *λ*, can be calculated as

1 $$ \lambda ={\displaystyle \sum_i{\displaystyle \sum_{j\ne i}{\pi}_i{q}_{ij}}}. $$

where *π*_*i*_ (*i* = T, C, A, or G) represents the equilibrium frequency of each of the four nucleotides. The probability of the nucleotide character changing from one nucleotide to another over a finite time period can then be described by a substitution probability matrix, **P**(*λ*, *t*), whose element *p*_*ij*_(*λ*, *t*) provides the probability that the character with average substitution rate *λ* will change from nucleotide *i* to nucleotide *j* (*j ≠ i*) after time *t*. The substitution probability matrix can be derived from the substitution rate matrix via the equation

2 $$ \mathbf{P}\left(\lambda, t\right)={\mathrm{e}}^{\mathbf{Q}\left(\lambda \right)t}. $$

**Figure 1:**
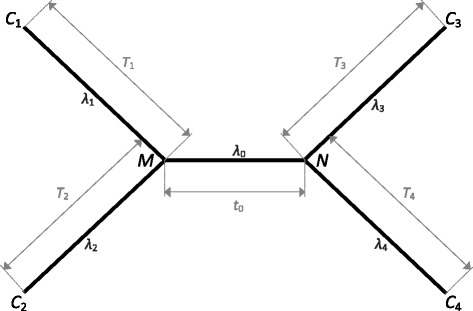
An unrooted four-taxon tree in an ultrametric form, with an internode of length (in time) *t*_0_ and four subtending branches of lengths (in time) *T*_1_, *T*_2_, *T*_3_, and *T*_4_. The ancestral states of a molecular character at the two ends of the internode are denoted as *M* and *N*. The character states at the terminal tips of the four subtending branches are denoted as *C*_1_, *C*_2_, *C*_3_, and *C*_4_. The average substitution rate of the character over the internode and the four subtending branches is denoted as *λ*_0_, *λ*_1_, *λ*_2_, *λ*_3_, and *λ*_4_. The expected number of character state changes in the internode and the four subtending branches are thus given by *λ*_0_ *t*_0_, *λ*_1_ *T*_1_, *λ*_2_ *T*_2_, *λ*_3_ *T*_3_, and *λ*_4_ *T*_4_, respectively.

Equation 2 can be solved via eigendecomposition (*c.f.* [57]). Using **P**(*λ*, *t*), we track the Markov chain of a nucleotide character in an ultrametric four-taxon tree with four uneven subtending branches. Let *M* and *N* denote the ancestral states of the nucleotide character at the two ends of the internode, whose length in time is represented by *t*_0_; let *C*_1_, *C*_2_, *C*_3_, and *C*_4_ represent the nucleotide character’s states at the terminal tips of the four subtending branches, whose lengths in time are denoted as *T*_1_, *T*_2_, *T*_3_, and *T*_4_, respectively (Figure 1). To allow unequal substitution rates of the character across the branches, we denote the average substitution rate of the character in the internode and the four subtending branches as *λ*_0_, *λ*_1_, *λ*_2_, *λ*_3_, and *λ*_4_, respectively (Figure 1).

The four-taxon tree has three possible tip-labeled subtrees, which we denote as *τ*_1_, *τ*_2_, and *τ*_3_, respectively; only one of the three subtrees (*τ*_3_) matches the actual quartet topology (*c.f.* Figure 1 in Townsend *et al.* [56]). Each of the three subtrees can be supported by an “AABB” pattern of character states (*i.e. τ*_3_ by *C*_1_ = *C*_2_ ≠ *C*_3_ = *C*_4_, *τ*_1_ by *C*_1_ = *C*_3_ ≠ *C*_2_ = *C*_4_, and *τ*_2_ by *C*_1_ = *C*_4_ ≠ *C*_2_ = *C*_3_ in Figure 1). A character exhibiting an AABB pattern that is consistent with the actual quartet topology (“synapomorphic pattern”, *i.e.C*_1_ = *C*_2_ ≠ *C*_3_ = *C*_4_ in Figure 1) contributes to correct resolution of the four-taxon tree, while a character showing an AABB pattern that is consistent with either of the two incorrect subtrees (“homoplasious pattern”, *i.e.C*_1_ = *C*_3_ ≠ *C*_2_ = *C*_4_, or *C*_1_ = *C*_4_ ≠ *C*_2_ = *C*_3_ in Figure 1) contributes to incorrect resolution of the tree. Summing the probabilities of all possible scenarios of character state changes across the internode and subtending branches that result in a desired pattern of character states at the four terminal tips as in Su *et al.* [57], the probability of a nucleotide character showing the synapomorphic pattern is provided by

$$ y\left({\lambda}_0,{\lambda}_1,{\lambda}_2,{\lambda}_3,{\lambda}_4;{t}_o,{T}_1,{T}_2,{T}_3,{T}_4\right) $$

3 $$ ={\displaystyle \sum_M{\displaystyle \sum_N{\displaystyle \sum_{C_1={C}_2}{\displaystyle \sum_{C_3={C}_4\ne {C}_1}{\pi}_M{p}_{MN}\left({\lambda}_0,{t}_0\right){p}_{M{C}_1}\left({\lambda}_1,{T}_1\right){p}_{M{C}_2}\left({\lambda}_2,{T}_2\right){p}_{N{C}_3}\left({\lambda}_3,{T}_3\right){p}_{N{C}_4}\left({\lambda}_4,{T}_4\right)}}}}. $$

Similarly, the probability of a character exhibiting either of the homoplasious patterns is provided by

4 $$ \begin{array}{l}{x}_1\left({\lambda}_0,{\lambda}_1,{\lambda}_2,{\lambda}_3,{\lambda}_4;{t}_o,{T}_1,{T}_2,{T}_3,{T}_4\right)\\ {}={\displaystyle \sum_M{\displaystyle \sum_N{\displaystyle \sum_{C_1={C}_3}{\displaystyle \sum_{C_2={C}_4\ne {C}_1}{\pi}_M{p}_{MN}\left({\lambda}_0,{t}_0\right){p}_{M{C}_1}\left({\lambda}_1,{T}_1\right){p}_{M{C}_2}\left({\lambda}_2,{T}_2\right){p}_{N{C}_3}\left({\lambda}_3,{T}_3\right){p}_{N{C}_4}\left({\lambda}_4,{T}_4\right)},}}}\end{array} $$

and

5 $$ \begin{array}{l}{x}_2\left({\lambda}_0,{\lambda}_1,{\lambda}_2,{\lambda}_3,{\lambda}_4;{t}_o,{T}_1,{T}_2,{T}_3,{T}_4\right)\\ {}={\displaystyle \sum_M{\displaystyle \sum_N{\displaystyle \sum_{C_1={C}_4}{\displaystyle \sum_{C_2={C}_3\ne {C}_1}{\pi}_M{p}_{MN}\left({\lambda}_0,{t}_0\right){p}_{M{C}_1}\left({\lambda}_1,{T}_1\right){p}_{M{C}_2}\left({\lambda}_2,{T}_2\right){p}_{N{C}_3}\left({\lambda}_3,{T}_3\right){p}_{N{C}_4}\left({\lambda}_4,{T}_4\right)}}}}.\end{array} $$

While the homoplasious patterns arise due to homoplasy (*i.e*. convergent state changes in non-sister subtending branches), the synapomorphic pattern can result from either true synapomorphy, or apparent synapomorphy due to homoplasy (*i.e.* parallel state changes in sister subtending branches [26,27,56]). The probability of true synapomorphy is characterized as the probability of a signal occurring in the internode (*i.e.* an informative difference in ancestral states at the two ends of the internode; corresponding to *M* ≠ *N* in Figure 1) multiplied by the probability of no subsequent state change in the four subtending branches. The probability of a signal occurring in the internode can be calculated by following a derivation similar to that presented in Equations 3-5, yielding

6 $$ \Pr \left\{\mathrm{a}\ \mathrm{difference}\ \mathrm{of}\ \mathrm{states}\ \mathrm{at}\ \mathrm{the}\ \mathrm{two}\ \mathrm{ends}\ \mathrm{of}\ \mathrm{the}\ \mathrm{internode}\right\}={\displaystyle \sum_M{\displaystyle \sum_{N\ne M}{\pi}_M{p}_{MN}\left({\lambda}_0,{t}_0\right)}}. $$

The probability of the signal remaining unobscured by subsequent state changes in the subtending branches can be evaluated by

7 $$ \Pr \left\{\mathrm{zero}\ \mathrm{state}\ \mathrm{changes}\ \mathrm{in}\ \mathrm{the}\ \mathrm{four}\ \mathrm{subtending}\ \mathrm{branches}\right\} = {\mathrm{e}}^{-\left({\lambda}_1{T}_1+{\lambda}_2{T}_2+{\lambda}_3{T}_3+{\lambda}_4{T}_4\right)} $$

(*c.f.* [27,60]). Thus, the probability of true synapomorphy is the product of Equations 6 and 7,

8 $$ \varPi \left({\lambda}_0,{\lambda}_1,{\lambda}_2,{\lambda}_3,{\lambda}_4;{t}_o,{T}_1,{T}_2,{T}_3,{T}_4\right)=\left({\displaystyle \sum_M{\displaystyle \sum_{N\ne M}{\pi}_M{p}_{MN}\left({\lambda}_0,{t}_0\right)}}\right){\mathrm{e}}^{-\left({\lambda}_1{T}_1+{\lambda}_2{T}_2+{\lambda}_3{T}_3+{\lambda}_4{T}_4\right)}. $$

The probability of apparent synapomorphy is thus provided by subtracting Equation 8 from Equation 3.

Note although the derivation of Equations 3–8 above is presented for nucleotide characters, these equations are also applicable to amino acid characters by substituting an amino acid substitution rate matrix for the nucleotide substitution rate matrix **Q**(*λ*) in Equations 1 and 2, and could also be applied to morphological characters that evolve in accord with the Mk matrix [61,62].

#### Predicting phylogenetic utility

To simplify notation hereafter, we will suppress the routine but continuing functional dependencies on *λ*_0_, *λ*_1_, *λ*_2_, *λ*_3_, *λ*_4_, *t*_0_, *T*_1_, *T*_2_, *T*_3_, and *T*_4_. Because parsimony uses almost exclusively the AABB patterns to inform quartet topology reconstruction, evaluating *y* − Max(*x*_1_, *x*_2_) for a molecular character gives an accurate quantitative measure of the character’s phylogenetic utility for resolving a quartet phylogeny as assessed by parsimony. For a given character, if *y* − Max(*x*_1_, *x*_2_) > 0, the character has more support for the correct quartet topology than for either of the incorrect quartet topologies as assessed by parsimony, and thus by sampling more of such a character, inference via parsimony will converge to the correct topology. Conversely, if *y* − Max(*x*_1_, *x*_2_) < 0, the character has a stronger support for an incorrect topology than for the correct topology as assessed by parsimony, and thus by sampling more of such a character, inference via parsimony will not converge to the correct topology. Therefore, evaluating *y* − Max(*x*_1_, *x*_2_) yields a quantitative measure of whether inference will be consistent under parsimony.

However, evaluating *y* − Max(*x*_1_, *x*_2_) for predicting phylogenetic utility and consistency conditions under probabilistic inference methods such as ML and Bayesian methods faces two opposing biases. First, ML and Bayesian methods can obtain additional information to resolve a quartet phylogeny—albeit of markedly lower impact per character—from some non-AABB patterns. For example, given a non-AABB pattern observed at a character that resulted from a signal in the internode having then been partially masked by noise (*i.e.* randomizing state changes in subtending branches), a probabilistic inference method will attribute likelihood to the correct topology from this character if the state changes that occurred in subtending branches are consistent enough with the model and occurred slowly enough to provide useful information. On the other hand, unlike with parsimony-based inference, not every character showing an AABB pattern is interpreted by probabilistic methods to support a quartet topology. For instance, given a synapomorphic pattern observed at a character that actually arose from an absence of state change in the internode followed by parallel state changes in sister subtending branches, a probabilistic method that classifies the site as fast-evolving will rightfully obtain little support for the correct topology from this character.

Addressing the first bias as outlined in the preceding paragraph is not straightforward within the framework of signal and noise analysis, because tracking all non-AABB patterns that can have varying and ambiguous levels of support for the correct quartet topology as assessed by probabilistic inference methods is impractical and would render analysis highly cumbersome. However, the second bias as explained above can be addressed by evaluating an alternative measure of predicted utility that excludes support for the correct quartet topology due to apparent synapomorphy. Such a measure can be obtained by comparing the probability of true synapomorphy only, *Π*, to the probability of observing either homoplasious pattern consistent with an incorrect quartet topology, Max(*x*_1_, *x*_2_). The resultant measure, *Π* − Max(*x*_1_, *x*_2_), represents a conservative lower bound of utility, since it does not include support for the correct quartet topology due to partially masked signal, which parsimony typically does not recognize but probabilistic inference methods can recognize under ideal circumstances. Ultimately, because true synapomorphy represents unmasked, actual phylogenetic signal and provides unambiguous support for the correct quartet topology regardless of which inference method is concerned, in branch length conditions where *Π* − Max(*x*_1_, *x*_2_) > 0, the strength of unmasked actual signal is greater than the strength of homoplasy that supports an incorrect topology, and therefore correct inference can likely be achieved by both parsimony and probabilistic methods.

## Results

### Example 1: predicted utility of a character in the felsenstein and “Farris” zones

In demonstrating long-branch attraction by parsimony and “long-branch repulsion” by ML, Huelsenbeck and Hillis [22] and Siddall [26] performed simulations for two four-taxon model trees with different branch length conditions that encompass the Felsenstein zone and the Farris zone, respectively. In this example study, we apply the signal and noise analysis to these two model trees to predict the phylogenetic utility of a nucleotide character in the Felsenstein zone and the Farris zone.

For this analysis, we assume the Jukes-Cantor (JC [63]) model—the simplest time reversible nucleotide substitution model—which both Huelsenbeck and Hillis [22] and Siddall [26] used in their respective simulation studies. To be consistent with Huelsenbeck and Hillis [22] and Siddall [26], we express the length of any tree branch, represented here as *p*, in terms of the expected probability that the nucleotide at one end of the branch differs from the nucleotide at the other end. Under the JC model, the *p* length of a branch can be related to the branch length in time, *t*, and the substitution rate of the nucleotide character in the branch, *λ*, via the equation

9 $$ p=\frac{3}{4}-\frac{3}{4}{\mathrm{e}}^{-\frac{4}{3}\lambda t}. $$

From Equation 9, the length of a branch can range between 0 and 0.75 under the JC model.

**Figure 2:**
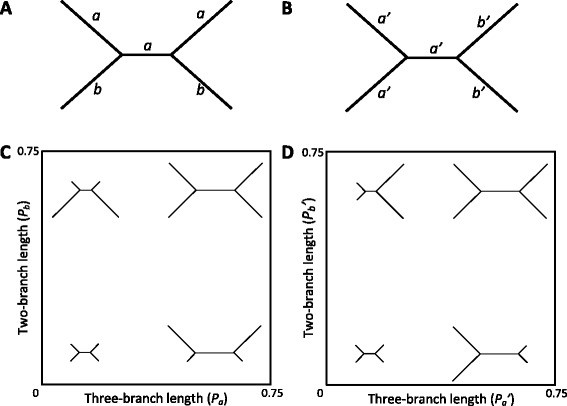
Two classic quartet branch length conditions in which long-branch effects can arise. **A)** Four-taxon tree modeled by Huelsenbeck and Hillis [22]. The internode and two subtending branches labeled *a* are constrained to have the same length (*i.e.* “three-branch length”), so are the two subtending branches labeled *b* (*i.e.* “two-branch length”); *p*_*a*_ and *p*_*b*_ represent the three-branch length and two-branch length (evaluated via Equation 9), respectively. **B)** Alternative four-taxon tree modeled by Siddall [26]. The internode and two subtending branches labeled *a’* are constrained to be equal in length (*i.e.* “alternative three-branch length”), so are the two subtending branches labeled *b’* (*i.e.* “alternative two-branch length”), with *p*_*a*_ *’* and *p*_*b*_ *’* representing the alternative three-branch length and two-branch length, respectively. **C)** Branch length space of the model tree investigated by Huelsenbeck and Hillis [22], with the three-branch length *p*_*a*_ on the horizontal axis and the two-branch length *p*_*b*_ on the vertical axis. These axes apply to Figures 3A, C, and E. The upper-left portion of this branch length space corresponds to the Felsenstein zone. **D)** Branch length space of the alternative model tree investigated by Siddall [26], with the alternative three-branch length *p*_*a*_ *’* on the horizontal axis and the alternative two-branch length *p*_*b*_ *’* on the vertical axis. These axes correspond to those in Figures 3B, D, and F. The upper-left portion of this branch length space corresponds to the Farris zone as termed by Siddall [26].

The four-taxon tree modeled by Huelsenbeck and Hillis [22] is shown in Figure 2A. The tree’s internode and two subtending branches on the opposite sides of the internode are constrained to be equal (“three-branch length”, *i.e. λ*_0_*t*_0_ = *λ*_1_*T*_1_ = *λ*_3_*T*_3_ in Figure 1), as are the other two subtending branches (“two-branch length”, *i.e. λ*_2_*T*_2_ = *λ*_4_*T*_4_ in Figure 1). Figure 2B shows the alternative four-taxon tree modeled by Siddall [26]. In this case, the internode and the two subtending branches on one side of the internode are constrained to be equal (*i.e. λ*_0_*t*_0_ = *λ*_1_*T*_1_ = *λ*_2_*T*_2_ in Figure 1), so are the two subtending branches on the other side of the internode (*i.e. λ*_3_*T*_3_ = *λ*_4_*T*_4_ in Figure 1). Figures 2C and D show the branch length space of the two model trees, each constructed by varying the respective tree’s three-branch length on the horizontal axis and two-branch length on the vertical axis. The Felsenstein zone is in the upper-left portion of the branch length space of the Huelsenbeck and Hillis [22] model tree, and the Farris zone is in the upper-left portion of the branch length space of the Siddall [26] model tree.

**Figure 3:**
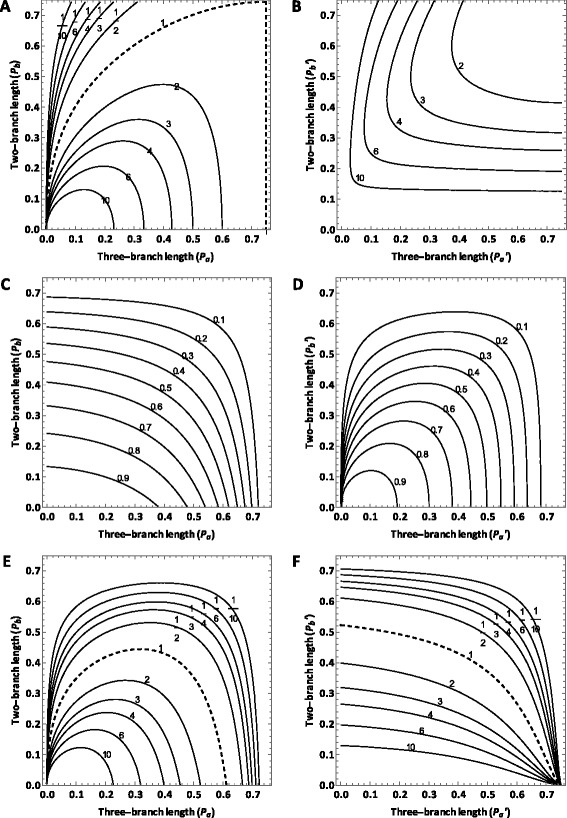
Contour map of *y* ∕ Max(*x*_1_, *x*_2_) for a nucleotide character which assumes the JC model over the branch length space of **A)** the Huelsenbeck and Hillis [22] model tree and **B)** the Siddall [26] model tree, with contour lines of *y* ∕ Max(*x*_1_, *x*_2_) = 1/10, 1/6, 1/4, 1/2, 1 (thick dashed), 2, 4, 6, and 10 shown if present within the respective branch length space. Contour map of *Π* ∕ *y* for a nucleotide character under the JC model over the branch length space of **C)** the Huelsenbeck and Hillis [22] model tree and **D)** the Siddall [26] model tree, with contour lines of *Π* ∕ *y* = 0.1, 0.2, 0.3, 0.4, 0.5, 0.6, 0.7, 0.8, 0.9, and 1.0 (thick dashed) shown if present. Contour map of *Π* ∕ Max(*x*_1_, *x*_2_) for a nucleotide character under the JC model over the branch length space of **E)** the Huelsenbeck and Hillis [22] model tree and **F)** the Siddall [26] model tree, with contour lines of *Π* ∕ Max(*x*_1_, *x*_2_) = 1/10, 1/6, 1/4, 1/2, 1 (thick dashed), 2, 4, 6, and 10 shown if present.

For the Huelsenbeck and Hillis [22] model tree, the probability of a nucleotide character showing the synapomorphic pattern is less than that of a homoplasious pattern (*i.e. y* ∕ Max(*x*_1_, *x*_2_) < 1) in an area located in the upper-left portion of the branch length space, which corresponds to the Felsenstein zone (Figure 3A). In contrast, for the Siddall [26] model tree, *y ∕* Max(*x*_1_, *x*_2_) > 1 is true in virtually the whole branch length space (Figure 3B). For both model trees, in the uppermost and rightmost areas of the branch length space, true synapomorphy accounts for less than 10% the probability of a character showing the synapomorphic pattern (*i.e.Π* ∕ *y* < 0.1) (Figures 3C and D). For the Siddall [26] model tree, *Π* ∕ *y* < 0.1 is also true in an additional area in the upper-left portion of the branch length space, which falls within the Farris zone (Figure 3D).

For the Huelsenbeck and Hillis [22] model tree, the probability of true synapomorphy is greater than the probability of a character exhibiting either homoplasious pattern (*i.e.Π* ∕ Max(*x*_1_, *x*_2_) > 1) in an area that borders on the horizontal axis of the branch length space (Figure 3E). For the Siddall [26] model tree, *Π* ∕ Max(*x*_1_, *x*_2_) > 1 is true in a similar but slightly more extended area that borders on both the horizontal and vertical axis of the branch length space (Figure 3F).

### Example 2: predicted utility of a character with an identical rate across lineages for resolving an asymmetrical quartet tree

In this example, we assess the predicted utility of a nucleotide character for resolving a hypothetical four-taxon tree with an asymmetrical topology. For this analysis we consider a nucleotide character which follows the molecular clock assumption and has an equal substitution rate in the internode and four subtending branches in the four-taxon tree of interest (*i.e.* setting *λ*_0_ = *λ*_1_ = *λ*_2_ = *λ*_3_ = *λ*_4_ = *λ* in Figure 1). We assume the JC model for the nucleotide character. The four-taxon tree in question has an internode with a length in an arbitrary time unit of *t*_0_ = 0.1; two non-sister subtending branches have an equal length of 4*t*_0_ = 0.4 (*i.e.* setting *T*_1_ = *T*_3_ = 0.4 in Figure 1), while the other two non-sister subtending branches both have a length of 0.4*l* (*i.e. T*_2_ = *T*_4_ = 0.4*l* in Figure 1), where *l* > 1.

**Figure 4:**
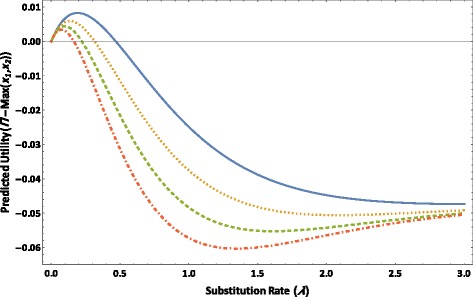
The predicted utility *Π* − Max(*x*_1_, *x*_2_) versus substitution rate *λ* based on the JC model is plotted for *l =* 1.5 (solid line), *l* = 2 (dotted line), *l* = 2.5 (dashed line), and *l* = 3 (dot-dashed line), for the four-taxon tree as depicted in Figure 1 in which *λ*_0_ = *λ*_1_ = *λ*_2_ = *λ*_3_ = *λ*_4_ = *λ*, *t*_0_ = 0.1, *T*_1_ = *T*_3_ = 0.4, and *T*_2_ = *T*_4_ = 0.4*l*.

The value of *Π* − Max(*x*_1_, *x*_2_) increases as a function of *λ* for each value of *l =* 1.5, 2, 2.5, and 3 for the four-taxon tree until reaching a maximum at an optimal substitution rate (Figure 4). As *λ* increases further, the value of *Π* − Max(*x*_1_, *x*_2_) begins to decrease and then drops to zero at a threshold substitution rate (Figure 4). As *λ* increases beyond that threshold, the value of *Π* − Max(*x*_1_, *x*_2_) becomes negative. Given each value of *l*, as *λ* increases from zero, the value of *Π* − Max(*x*_1_, *x*_2_) increases from zero. As the value of *l* increases, corresponding to an increasingly asymmetrical topology, the maximum value of *Π* − Max(*x*_1_, *x*_2_) decreases as do the optimal and threshold substitution rates.

### Example 3: predicted utility of a character with a variable rate across lineages for resolving a symmetric quartet tree

In this example, we evaluate the predicted utility of a nucleotide character for resolving a hypothetical four-taxon tree with a symmetric topology. The four-taxon tree in question has an internode with a length (in time) of *t*_0_ = 0.1 and four subtending branches with an equal length of 0.1*l*, where *l* > 1 (*i.e.* setting *T*_1_ = *T*_2_ = *T*_3_ = *T*_4_ = 0.1*l* in Figure 1). For this analysis, we again assume the JC model for the nucleotide character; however, the character does not necessarily follow a molecular clock across the quartet. We assign a fixed substitution rate of 1 (per unit time) to two non-sister subtending branches of the four-taxon tree (*i.e. λ*_2_ = *λ*_4_ = 1 in Figure 1), and a free substitution rate of *λ* in the internode and the other two non-sister subtending branches of the tree (*i.e.* setting *λ*_0_ = *λ*_1_ = *λ*_3_ = *λ* in Figure 1).

**Figure 5:**
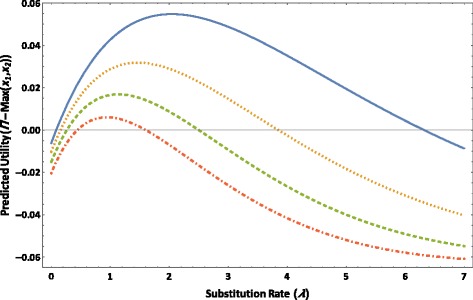
The predicted utility *Π* − Max(*x*_1_, *x*_2_) versus substitution rate *λ* based on the JC model is plotted for *l* = 1.5 (solid line), *l* = 2 (dotted line), *l* = 2.5 (dashed line), and *l* = 3 (dot-dashed line), for the four-taxon tree as depicted in Figure 1 in which *t*_0_ = 0.1, *T*_1_ = *T*_2_ = *T*_3_ = *T*_4_ = *lt*_0_, *λ*_1_ = *λ*_3_ = 1, and *λ*_0_ = *λ*_2_ = *λ*_4_ = *λ*.

The value of *Π* − Max(*x*_1_, *x*_2_) as a function of *λ* starting from *λ* = 0 first increases from a negative value until reaching a positive maximum at an optimal rate (Figure 5), across values of *m* = 1.5, 2, 2.5, and 3 for the four-taxon tree. It then decreases monotonically as *λ* increases beyond the optimal rate. Given each value of *m*, the value of *Π* − Max(*x*_1_, *x*_2_) is positive and close to its maximum when the substitution rate of the character is similar in the four subtending branches (*i.e.* when *λ* is close to 1). As the value of *m* increases, corresponding to an increasingly deep internode, the maximum value of *Π* − Max(*x*_1_, *x*_2_) decreases, and so do the optimal rate of *λ* and the range of parameter *λ* for which the value of *Π* − Max(*x*_1_, *x*_2_) is positive.

### Example 4: effects of alternative model assumptions on predicted utility

Su *et al.* [57] evaluated the impact of specifying alternative nucleotide substitution models on the predicted utility of nucleotide characters for resolving a four-taxon tree with even subtending branches, based on an analysis of five genes in 29 taxa of the yeast genus *Candida* and allied teleomorph genera. Similarly, here we compare how varying the model specification affects the predicted utility of a nucleotide character for resolving a four-taxon tree with uneven subtending branches due to unequal substitution rates. We perform this analysis based on the nucleotide character and the hypothetical four-taxon tree as used in Example 3 above, with *m* = 2.5 for the four-taxon tree (*i.e.* setting *λ*_1_ = *λ*_3_ = 1, *λ*_0_ = *λ*_2_ = *λ*_4_ = *λ*, *t*_0_ = 0.1, and *T*_1_ = *T*_2_ = *T*_3_ = *T*_4_ = 0.25 in Figure 1). We assume four alternative nucleotide substitution models for the nucleotide character, including—from simple to complex—the JC model, which assumes equal substitution rates and equal base frequencies at equilibrium, the Kimura 2-Parameter (K2P *a.k.a.* K80 [64]) model, which assumes unequal transition and transversion rates and equal base frequencies, the Hasegawa-Kishino-Yano (HKY [65]) model, which assumes unequal transition and transversion rates and unequal base frequencies, and the GTR model, which assumes six unequal substitution rates and unequal base frequencies (*c.f.* Table 1 in [57]). The parameter values for the JC, K2P, HKY, and GTR models used in this analysis are based on the parameter values of these models estimated for the actin (ACT1) marker in the analysis by Su *et al.* [57] of 29 taxa of the yeast genus *Candida* and allied teleomorph genera (Table 1).

**Table 1:** **Estimated parameter values for the models for the actin (ACT1) marker**

	**JC**	**K2P**	**HKY**	**GTR**
**rTC**	1	4.493	4.522	9.082
**rTA**	1	1	1	1.967
**rTG**	1	1	1	1
**rCA**	1	1	1	1.078
**rCG**	1	1	1	0.907
**rAG**	1	4.493	4.522	2.902
**πT**	0.25	0.25	0.336	0.265
**πC**	0.25	0.25	0.274	0.225
**πA**	0.25	0.25	0.235	0.286
**πG**	0.25	0.25	0.155	0.224

**Figure 6:**
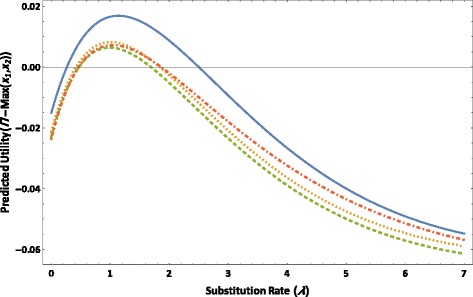
The predicted utility *Π* − Max(*x*_1_, *x*_2_) versus substitution rate *λ* is plotted based on the JC [63] model (solid line), the K2P (dotted line), the HKY (dashed line), and the GTR model (dot-dashed line), for the four-taxon tree as depicted in Figure 1 in which *t*_0_ = 0.1, *T*_1_ = *T*_2_ = *T*_3_ = *T*_4_ = 0.25, *λ*_1_ = *λ*_3_ = 1, and *λ*_0_ = *λ*_2_ = *λ*_4_ = *λ*. The model parameter values are presented in Table 1.

The value of *Π* − Max(*x*_1_, *x*_2_) of the character as a function of *λ* is highest under the JC model (Figure 6). The range of the parameter *λ* within which *Π* − Max(*x*_1_, *x*_2_) is positive is wider under the JC model than under the three higher parameterized models; this range differs little among the K2P, HKY, and GTR models.

## Discussion

In this paper, we have relaxed an assumption of phylogenetic signal and noise analysis by allowing a four-taxon tree of unequal subtending branch lengths. Previous analyses [56,57] assumed a phylogenetic quartet with four subtending branches of equal lengths. Although any internode has an inherent quartet structure [66], not all internodes have subtending branches that have equal lengths, even without heterochrony. Furthermore, sampling additional taxa can effectively reduce branch lengths [67-72], rendering appropriate branch lengths to consider for phylogenetic informativeness shorter than the extracted quartet. While slight differences in branch lengths probably do not represent a significant violation of the theoretical assumption under the previous versions of signal and noise analysis, for internodes where all of the subtending branches have markedly different lengths, the assumption of equal branch lengths is no longer acceptable. The generality and the accuracy of the signal and noise analysis can therefore be improved by quantifying the probability of synapomorphic and homoplasious character state patterns in four subtending branches of unequal lengths. This improvement, if it could seamlessly incorporate increased taxon sampling in addition, would facilitate the application of signal and noise analysis freely and precisely to all describable internodes of phylogenetic interest.

We have also recast previous analysis so that it can characterize the probability of a true synapomorphy in a four-taxon tree, including only true synapomorphy as support for the correct quartet topology. Previous signal and noise analyses [56,57] have not distinguished true synapomorphy vs. apparent synapomorphy and include both as support for the correct quartet topology. While parsimony infers support for the correct quartet topology from both true synapomorphy and apparent synapomorphy, probabilistic inference methods can better discriminate against apparent synapomorphy by accounting for fast rates of evolution and correcting for unobserved changes [6,27,28,73]. In the meantime, however, the generalized signal and noise analysis does not quantify contributions from obscured signal at sites that are not parsimony-informative, even though probabilistic inference methods can recognize some support for the correct topology from these sites. Therefore, including support for the correct quartet topology only from true, unobscured parsimony-informative sites yields a conservative lower bound for predicting phylogenetic utility.

In the first example, based on the two model quartet trees with branch length conditions that correspond to the Felsenstein and “Farris” zones, our analysis has characterized the probability distributions of true synapomorphy, apparent synapomorphy, and homoplasy in support for an incorrect topology in the those zones. These analysis results provide analytical predictions of the contrasting performances of parsimony and ML in the Felsenstein and Farris zones as shown by simulations of Huelsenbeck and Hillis [22] and Siddall [26]. In the Felsenstein zone, parsimony is likely to give incorrect inference of the quartet topology, because support for the correct quartet topology as assessed by parsimony (*i.e.* including both true and apparent synapomorphy) is less than support for an incorrect topology in the corresponding area of the branch length space. This observation is consistent with the expectation that parsimony-informative sites that are consistent with an incorrect quartet topology are more likely to occur and accumulate if the internode is short (*i.e.* there is a low probability of true signal occurring in the internode), the rate of evolution of the character is fast (*i.e.* there is a high probability of noise accumulating in the subtending branches), or the differences in the rate of evolution between branches is large (*i.e.* there is a high probability of convergent and parallel changes in the two non-sister branches with faster rates of evolution). In contrast, ML can perform better than parsimony by gathering additional support for the correct quartet topology from partially-informative non-AABB patterns, which are not tracked by our theory. In the Farris zone, parsimony is likely to yield correct inference of the quartet topology, since support for the correct quartet topology as assessed by parsimony is greater than support for either incorrect topology in the corresponding area of the branch length space. However, the strong performance of parsimony in the Farris zone is in fact due to apparent synapomorphy; in the corresponding area of the branch length space, almost all support for the correct quartet topology is contributed to by apparent synapomorphy. Since ML does not accrue likelihood for the correct quartet topology in the presence of apparent synapomorphy in the way that parsimony does, ML is not misled into performing as well as parsimony in the Farris zone in terms of recovering the correct quartet topology.

This generalized signal and noise analysis can be applied to diverse scenarios in which unequal branch lengths can arise and potentially introduce long-branch effects. Unequal branch lengths can be either caused by unequal evolution rates across lineages within the study group (*i.e.* relaxation of the molecular clock assumption), or due to an asymmetrical topology, which can arise as a result of differential speciation or extinction rates and/or incomplete taxon sampling [6]. The signal and noise theory decouples the rate of substitution and time in characterizing the length of a branch. Thus, the theory can account for differences in both substitution rates and evolution times across lineages, and it can be applied to phylogenies in which unequal branch lengths occur due to unequal rates of evolution, asymmetrical topologies, or both.

In the second example, based on a four-taxon tree with an asymmetrical topology, results of the signal and noise analysis demonstrated that the chance of correctly resolving an asymmetrical quartet phylogeny can be increased by sampling slower-evolving molecular loci; the more asymmetrical the underlying topology is, the slower-evolving the sampled molecular loci should be. Rapidly-evolving molecular loci have poor predicted phylogenetic utility because at these loci, there is a higher probability of observing noise or homoplasy than actual signal. For the quartet tree used in this example study, the signal and noise analysis furthermore quantified the threshold substitution rate above which a nucleotide character may contribute a negative utility towards correct resolution of the quartet tree. In molecular phylogenetic investigations, a common practice to reduce long-branch effects is to exclude fast-evolving molecular loci—such as third codon positions—from inference analysis, based on the rationale that these loci are likely saturated or randomized [19,40,74-80]. On the other hand, third codon positions can contain a significant amount of information of the phylogenetic structure [81], and removing an excessive amount of rapidly-evolving loci can lead to a significant reduction in resolution [79,80,82]. Therefore, for an actual quartet phylogeny for which the inferred topology is suspected to result from long-branch effects, by applying the generalized signal and noise analysis to an alternative topology that is hypothesized to reflect the actual taxon relationship, one can estimate a threshold substitution rate for sampling molecular loci for overcoming the suspected long-branch effects while in the meantime minimizing the number of fast-evolving loci that are unnecessarily excluded from analysis.

In the third example, in which the substitution rate of a nucleotide character was variable across the four taxa within the study group, the signal and noise analysis demonstrated that in addition to sampling slower-evolving molecular loci, sampling loci with less variation in substitution rate across lineages is helpful for avoiding biases towards topologies that group faster-evolving non-sister branches together. The deeper the internode in question is, the more likely there is to be significant rate variation, and yet the deeper the internode is, the less variation in substitution rate across lineages the sampled molecular loci should have. At molecular loci with significant rate variation across lineages, convergent or parallel character state changes tend to accumulate along the lineages with faster substitution rates, thereby obscuring actual signal and reducing the phylogenetic utility of these loci. For the quartet tree assessed in this example, the signal and noise analysis has also quantified the range of rate variation across lineages within which a nucleotide character has a positive predicted utility towards correct quartet resolution. In phylogenetic studies, another proposed approach to reducing long-branch effects involves selecting only representative taxa with the lowest substitution rates and minimum rate variation across lineages [83-85]. However, numerous studies have suggested that increased taxonomic sampling generally leads to improved accuracy in phylogenetic inference ([67,68,75,86-90]; but see also [3,91]; as summarized in [6,7]), and excluding a large number of taxa may thus significantly decrease the accuracy of inference outcomes. Therefore, in an investigation in which the inferred topology is suspected to arise due to long-branch effects, by applying the generalized signal and noise analysis to an alternative topology hypothesized to reflect the actual taxon relationship, one may estimate the desirable range of rate variation across lineages to inform taxon sampling while at the same time avoiding removing an excessive number of taxa from analysis.

Lastly, in the fourth example, which compared utility prediction for the four-taxon tree in the previous example based on four alternative nucleotide substitution models (*i.e.* the JC, K2P, HKY, and GTR models), analysis results indicated that predictions of the signal and noise analysis are fairly robust to alternative model specifications, consistent with the finding of Su *et al.* [57] in quartet trees with even subtending branches. In this example based on a four-taxon tree with unequal substitution rates across lineages, the predicted utility is higher under the JC model than under the other three more complex models; but as the model parameterization increases from the K2P model to the GTR model, the predicted utility remains largely unchanged. As explained by Su *et al.* [57], in most realistic molecular data sets, there is always a certain degree of heterogeneity in model parameter values when the data are fitted to an optimal model. As the model grows in complexity, some character states, due to association with higher model parameter values, will begin to dominate the evolutionary process and thus effectively reduce the character state space. Analysis results of Su *et al.* [57] also demonstrated that the predicted utility of a molecular character increases as the character state space increases (*c.f.* Figure 6 in [57]). Thus, specifying an overly simple model can fail to adequately account for heterogeneity in the evolutionary process and hence cause an increase of the effective character state space. Consequently, the predicted utility based on an overly simple model is higher than actual. But once a model of sufficient complexity is fitted to the molecular data in question, the effective character state space is reduced closer to its actual size, and the predicted utility is more accurate. Therefore, specifying increasingly more complex models will lead to decreasingly little impact on predictions of the signal and noise analysis.

## Conclusion

In this paper, we have generalized phylogenetic signal and noise analysis by allowing a four-taxon tree of unequal subtending branch lengths. This generalized signal and noise analysis provides analytical prediction of utility of characters evolving at diverse rates of evolution to resolve quartet phylogenies in which unequal branch lengths arise due to unequal rates of evolution, asymmetrical topologies, or both.

## Methods

Results and figures presented in the Result section were obtained by implementing the analytical calculations as outlined in the Theory section via Wolfram Mathematica 7 (Wolfram Research, Inc.).

### Research ethics

Research ethical approval and consent are not applicable to this study, since the study involves no human subjects.

## Abbreviations

GTR: General Time Reversible
HKY: Hasegawa, Kishino, and Yano
JC: Jukes and Cantor
K2P: Kimura 2-Parameter
ML: Maximum Likelihood
RASA: Relative Apparent Synapomorphy Analysis
